# Data on the predictions of plant redistribution under interplays among climate change, land-use change, and dispersal capacity

**DOI:** 10.1016/j.dib.2022.108667

**Published:** 2022-10-13

**Authors:** Kyung Ah Koo, Seon Uk Park

**Affiliations:** a Korea Environment Institute, 370 Sicheong-daero, Sejong-si 30147, Republic of Korea; b Div. of Restoration Strategy, National Institute of Ecology, Yeongyang 36531, Republic of Korea

**Keywords:** Plant redistribution, Climate change, Land-use change, Species distribution model (SDM)-Dispersal-Land-use change modeling

## Abstract

The future distribution data of *Pittosporum tobira, Raphiolepis indica* var. *umbellata, Neolitsea sericea, Ilex integra*, and *Eurya emarginata* were acquired from the MigClim, a GIS-based (hybrid) cellular automation model, modeling and the traditional SDM modeling using BioMod2. The current SDM projections, the traditional SDM predictions, which were assumed the climate-change-only, and model validation were performed using BioMod2 with 686 presence/absence data for each plant species. The MigClim predictions were performed under the combination of two climate change scenarios (RCP 4.5 and RCP 8.5), two land-use change scenarios (SSP1 and SSP3), and four dispersal scenarios (no dispersal, short-distance dispersal, long-distance dispersal, and full dispersal). For the MigClim predictions, the initial distribution map was produced by coupling the current land-use map with the ensemble SDM predictions for each plant. The future habitat suitability map was predicted by coupling the land-use prediction with the SDM predictions under RCP 4.5 and RCP 8.5. For the land-use map, the future land-use maps were predicted under SSP1 and SSP3 using the Integrated Valuation of Ecosystem Services and Tradeoffs (InVEST) Scenario Generator tool, and the land-use categories were classified into two classes, namely barrier and non-barrier. The degree of dispersal for each species was calculated using a negative exponential function, where the coefficients were 0.005 (∼1 km) and 0.0005 (∼10 km). The future expansion of range was predicted through dispersal simulations of 80 times from 1990 to 2070. The prediction and analyzed data provide essential information and insight for understanding the climate change effects on the warm-adapted plants in interactions with land-use change and the dispersal process. These data can be used for detecting restoration areas for increasing connectivity among habitats, establishing protected areas, and developing environmental policies related to restoration and conservation.

**Specifications Table**

**Table:** 

Subject	Environmental Science
Specific subject area	Ecology and Ecological Modeling
Type of data	Table Graph Figure
How the data were acquired	The current and future species distribution maps predicted under only climate change were acquired by species distribution modeling using the BioMod2 R package. The maps of land-use changes were predicted using the Integrated Valuation of Ecosystem Services and Tradeoffs (InVEST) Scenario Generator tool. The future species distribution maps under climate change, land-use change, and dispersal capacity were predicted using MigClim, a GIS-based (hybrid) cellular automation model.
Data format	Raw and Analyzed
Description of data collection	686 presence/absence data for the SDM modeling were obtained from Koo (2001), Lee and Lim (2002), and the Korea National Arboretum. Climate data, the current and future bioclimatic data, were acquired from the WorldClim dataset. The raster land-use map was obtained from the National Environmental Information Network System.
Data source location	The presence/absence data of each species: Koo [4] and the Korea National Arboretum Climate data, the current and future bioclimatic data: WorldClim 2 dataset The raster land-use map: The National Environmental Information Network System
Data accessibility	Repository name: the Korea National Arboretum Direct URL to data: http://www.nature.go.kr/index.jsp Repository name: WorldClim version 2 Direct URL to data: http://www.worldclim.org/ Repository name: NEINS Direct URL to data: https://www.neins.go.kr/Index Species presence/absence data: https://data.mendeley.com/datasets/w9dr6rms6r The TSS and AUC evaluation result data: https://data.mendeley.com/datasets/hzv8ph3gfn The results of the model projections under the climate-change-only scenario: https://data.mendeley.com/datasets/392spt7yzm The image data including the 80 future projections and the five current projections: https://data.mendeley.com/datasets/6cf687vhgx
Related research article	K. Koo, S. Park, The effect of interplays among climate change, land-use change, and dispersal capacity on plant redistribution. Ecol. Ind. 142 (2022) 109192. https://doi.org/10.1016/j.ecolind.2022.109192

## Value of the Data

• The prediction and analyzed data provide essential information and insight for understanding the climate change effects on the warm-adapted plants in interactions with land-use change and the dispersal process.
• The prediction and analyzed data will accelerate knowledge for conservation management and plans and in all climate change-related research communities.
• These data can be used for detecting restoration areas for increasing connectivity among habitats, establishing protected areas, and developing environmental policies related to restoration and conservation.

## 1 Data Description

**Fig. 1:**
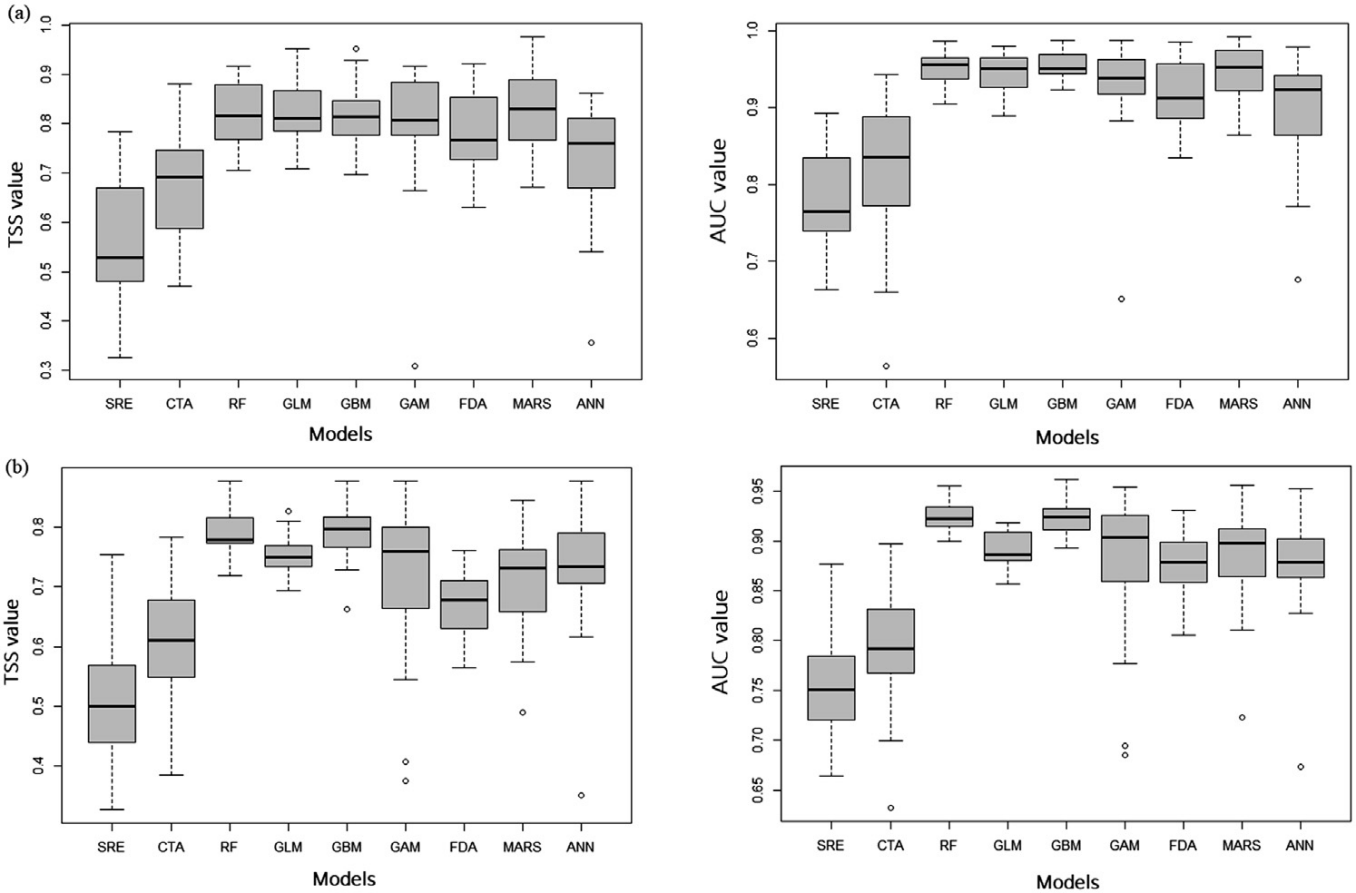

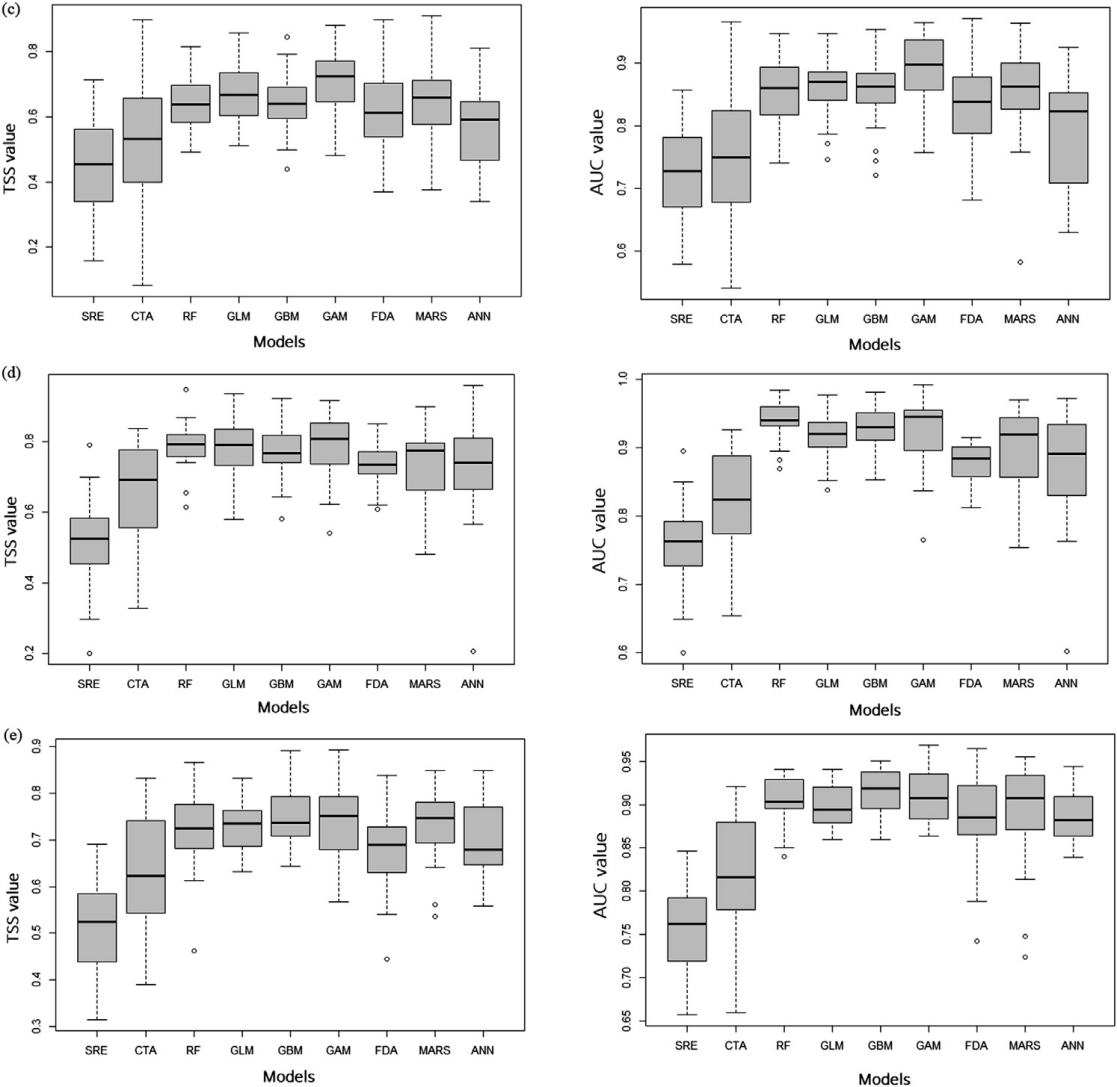
The TSS and AUC evaluation results of SDM predictions for *R. indica* var. *umbellate* (Fig. 1(a)), *N. sericea* (Fig. 1(b)), *I. integra* (Fig. 1(c)), *E. emarginata* (Fig. 1(d)), and *P. tobira* (Fig. 1(e)).

**Fig. 2:**
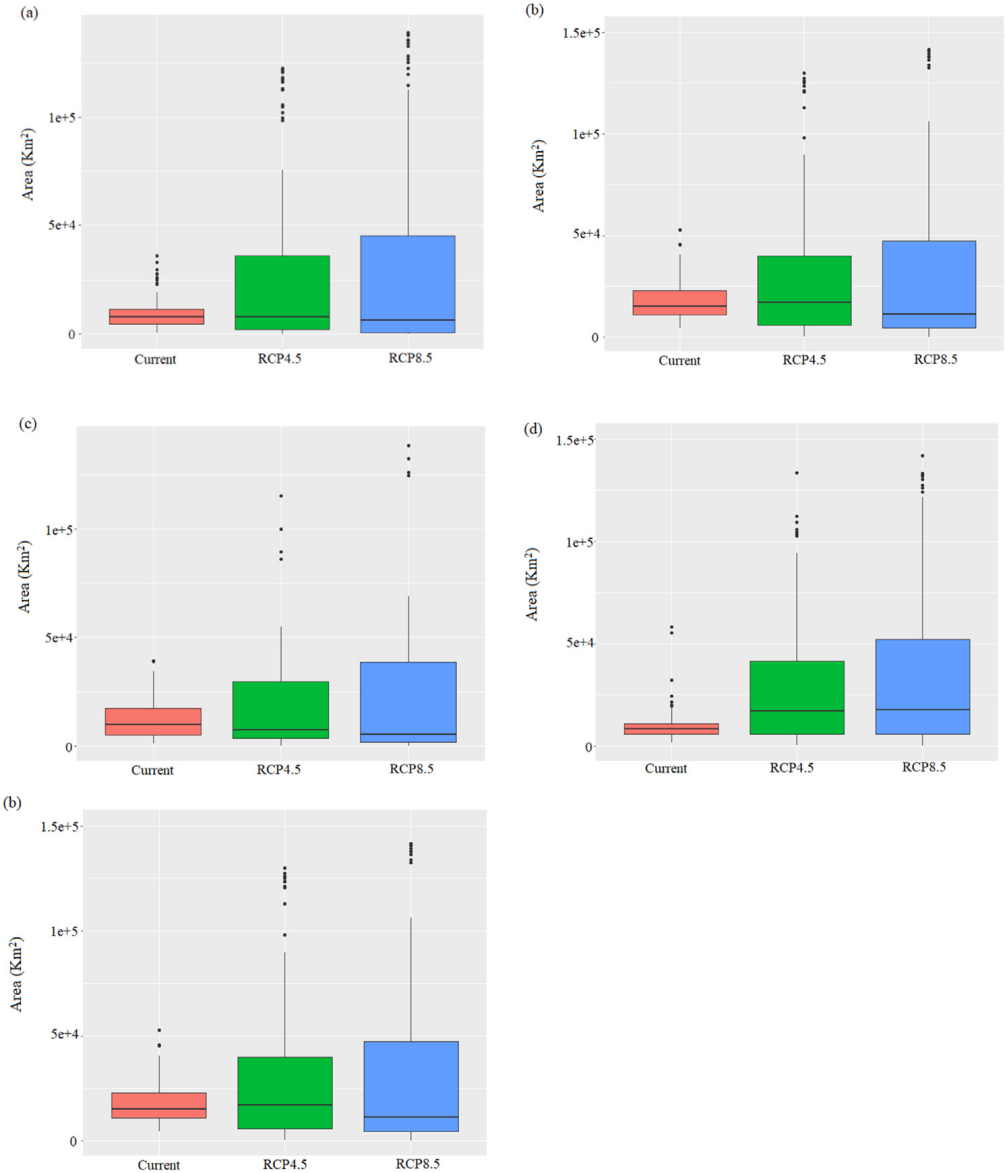
The results of the model projections and predictive uncertainty for the future distributions of five plants, *R. indica* var. *umbellate* (Fig. 2(a)), *N. sericea* (Fig. 2(b)), *I. integra* (Fig. 2(c)), *E. emarginata* (Fig. 2(d)), and *P. tobira* (Fig. 2(e)), predicted under the climate-change-only scenario.

Fig. 1 presents the range of TSS and AUC values of each model calculated for the ensemble SDM of five plant species, *Pittosporum tobira, Raphiolepis indica* var. *umbellata, Neolitsea sericea*, *Ilex integra*, and *Eurya emarginata*, and Fig. 2 the future distributions predicted under the climate-change-only scenario. Fig. 1 shows the model uncertainty in SDM projections and Fig. 2 the predictive uncertainly for the future distributions of five plants originated from different models and scenarios. Figs. 1 and 2 were predicted using the BioMod2 R package. For the SDM modeling, we used 686 presence/absence data for each plant species collected in the ROK. The 686 data were selected by removing data points close to each other, mostly < 5 km, to avoid violation of the general statistical modeling assumption originated from spatial autocorrelations among data and keeping a distance > 5 km between two points. It was exceptional, but the distance of a few data sampling points was between 2 km and 5 km. It was because the lists of plants of sampling points were totally different due to the difference of landscape, micro climates, etc. Therefore, the distances between the survey points were >2 km, mostly >5 km. Some data with inaccurate location information were also deleted from our dataset. It was a long-term national project of Korea National Arboretum (KNA) to build nation-wide forest species inventory. In this project, complete tree plant lists were surveyed at each data point; therefore, the survey points where no study species appeared were used as absence data. The number of species locations is 76 for *R. indica* var. *umbellate*, 77 for *N. sericea*, 54 for *I. integra*, 66 for *E. emarginata*, and 112 for *P. tobira*. These data are presented in supplementary files, deposited in the Mendeley database (https://data.mendeley.com/datasets/w9dr6rms6r). The data for the Fig. 1 is presented in supplementary files, deposited in the Mendeley database (https://data.mendeley.com/datasets/hzv8ph3gfn) and Fig. 2 in the supplementary files, deposited in the Mendeley database (https://data.mendeley.com/datasets/392spt7yzm).

**Fig. 3:**
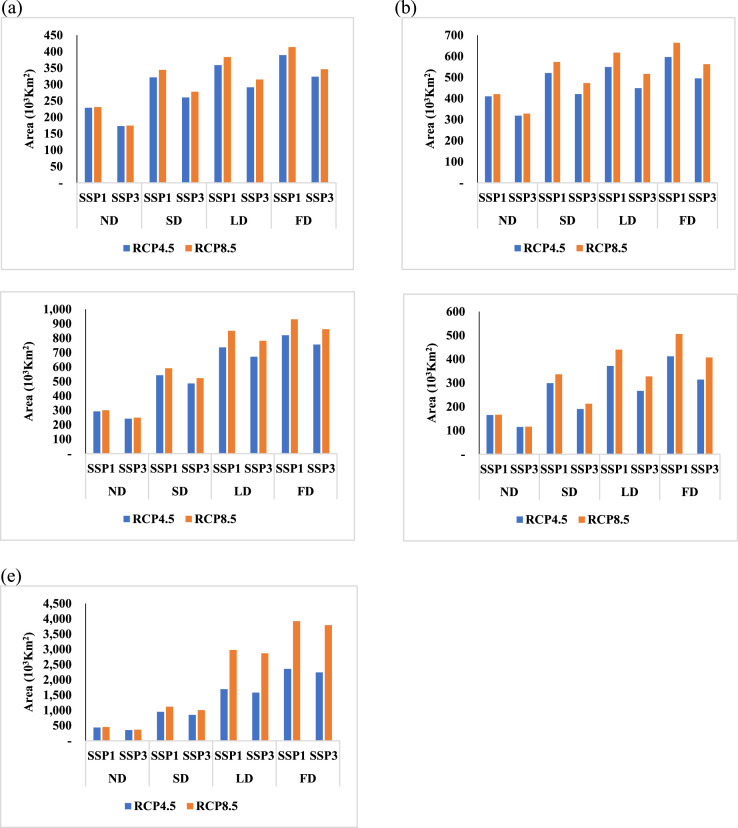
The future distribution areas of five plants, *R. indica* var. *umbellate* (Fig. 3(a)), *N. sericea* (Fig. 3(b)), *I. integra* (Fig. 3(c)), *E. emarginata* (Fig. 3(d)), and *P. tobira* (Fig. 3(e)) under combinations of climate changes (RCP 4.5 and RCP 8.5), land-use changes (SSP1 and SSP3) and dispersal capacity (ND, FD, SDD, and LDD).

Table 1 shows the predicted area of each land cover category under SSP scenarios, SSP1 and SSP 3. For the predictions, we assumed two land-use change scenarios, shared socioeconomic pathways (SSPs, SSP 1, and SSP 3). Fig. 3 shows the future distributional areas of five plant species under climate change, land-use change, and dispersal capacity predicted using the MigClim R package. For the predictions, we assumed two climate change scenarios, representative concentration pathways (RCPs, RCP 4.5, and RCP 8.5), two land-use change scenarios, shared socioeconomic pathways (SSPs, SSP 1, and SSP 3), and four dispersal scenarios, no dispersal (ND), short-distance dispersal (SDD), long-distance dispersal (LDD), and full dispersal (FD). The simulation produced the total 80 projections with 80 image files consisting of the 16 projections data of each plant. The image data including the 80 future projections and the five current projections (the initial maps for the simulations) are presented in supplementary files, deposited in the Mendeley database (https://data.mendeley.com/datasets/6cf687vhgx).

**Table 1:** Future areas of each land cover category under land-use change, which presents the projected areas under SSP scenarios, SSP1 and SSP 3. The unit of area is Km^2^.

Land Cover	Current	2070 (SSP1)	2070 (SSP3)
Urban area	413,060	493,353	740,046
Agricultural land	2,113,769	2,087,975	1,969,087
Forest	6,840,315	6,785,816	6,609,322
Grassland	285,632	285,632	285,632
Wetland	26,345	26,345	26,345
Bare land	161,134	161,134	209,823
Open waters	183,192	183,192	183,192

## 2 Experimental Design, Materials and Methods

The current SDM projections; the traditional SDM predictions, which assumed the climate-change-only; and model validation were performed using BioMod2 with 686 presence/absence data for each plant species. The model performances of SDMs were evaluated using the true skill statistics (TSS) and the area under the curve (AUC) statistics [1]. For the realistic predictions of species' future distribution, MigClim, a GIS-based (hybrid) cellular automation model, links the dispersal process and the land-use change to the SDM projection [2].

For MigClim predictions, the initial distribution map, which showed cells occupied by the species, was produced by coupling the current land-use map with the ensemble SDM predictions for each plant. The future habitat suitability map was predicted by coupling the land-use prediction with the SDM predictions under RCP 4.5 and RCP 8.5. For the land-use map, the future land-use maps were predicted under SSP1 and SSP3, and the categories were classified into two classes, namely barrier and non-barrier. Each class of barrier included urban area, agricultural land, wetland, bare land, open water, and non-barrier forest and grassland. We used the Integrated Valuation of Ecosystem Services and Tradeoffs (InVEST) Scenario Generator tool for the predictions [3]. For dispersal parameters, the degree of dispersal for each species was calculated using a negative exponential function. The coefficients of the functions were 0.005 (∼1 km) and 0.0005 (∼10 km). The future expansion of range was predicted through dispersal simulations of 80 times from 1990 to 2070.

The 686 presence/absence data of each species were obtained from the previous studies by Koo [4] and the Korea National Arboretum (http://www.nature.go.kr/index.jsp). We acquired the current and future bioclimatic data of BIO1, BIO2, BIO3, BIO12, BIO13, and BIO14 from the WorldClim 2 dataset (http://www.worldclim.org/), with a 30-arc-second (ca. 1 km^2^) spatial resolution. We used the future bioclimatic data predicted under RCP4.5 and RCP8.5 using the HadGEM-ES global circulation model.

## Ethics Statement

This study does not involve any modern human or animal subject.

## CRediT Author Statement

**Kyung Ah Koo:** Conceptualization, Methodology, Formal Data analysis, Project administration, Resources, Writing – original draft preparation, Reviewing & editing, Funding acquisition; **Seon Uk Park:** Resources, Simulation, Visualization, Validation.

## Declaration of Competing Interest

None.

## Data Availability

Species presence/absence data (Original data) (Mendeley Data). Koo&Park_ The image files of future projections for five evergreens (Original data) (Mendeley Data). Koo&Park_ The future distribution areas of five evergreens under climate change only (Original data) (Mendeley Data). Koo&Park_TSS&AUC evaluation results of SDM modeling (Original data) (Mendeley Data).
